# Incidence of Traumatic Diaphragmatic Injury: Results From a Retrospective Cohort Study in a Level I Trauma Center in Riyadh

**DOI:** 10.7759/cureus.47215

**Published:** 2023-10-17

**Authors:** Ibrahim Al Babtain, Bashayer AlObaid, Reema Alsogair, Maha A Aljrayed, Ghadi Almohaisen, Abrar Al-Mutairi

**Affiliations:** 1 Department of General Surgery, King Abdulaziz Medical City, Riyadh, SAU; 2 College of Medicine, King Saud Bin Abdulaziz University for Health Sciences, Riyadh, SAU; 3 College of Applied Medical Sciences, King Saud Bin Abdulaziz University for Health Sciences, Riyadh, SAU

**Keywords:** diaphragmatic rupture, traumatic diaphragmatic rupture, traumatic diaphragmatic hernia (tdh), traumatic diaphragmatic injuries, diaphragm injury

## Abstract

Introduction

Traumatic diaphragmatic injuries (TDIs) are uncommon and the incidence of TDI is difficult to estimate because of the variation in reporting missed or late detected diaphragmatic injuries. Therefore, our study’s aim was to investigate the prevalence of traumatic diaphragmatic injury in the thoracoabdominal trauma, discuss the etiological factors, diagnostic investigations, and outcomes in TDIs, and evaluate predictors of mortality in patients who were diagnosed at King Abdulaziz Medical City in Riyadh, Saudi Arabia.

Materials and methods

This observational retrospective cohort study was conducted at King Abdulaziz Medical City (KAMC), a tertiary hospital in Riyadh, Saudi Arabia. The study included all adult patients aged 18 years or older diagnosed with traumatic diaphragmatic injuries between the years 2016 and 2020. The BESTCare electronic system was used to get the patient's medical records and extract the data. Pearson χ2 test was used for categorical variables, and an independent t-test was used for continuous variables to investigate the association between predictors and outcomes.

Results

A total of eight patients were involved in this study. The mean age of the patients was 49 years old. Males outnumbered females by 75%. Patients admitted with blunt injuries were greater by 75% compared to penetrating injuries by 25%. The left side of the diaphragm was the most common site of injury. The total number of patients who were admitted to the ICU was five, four of whom had blunt trauma and one had a penetrating injury.

Conclusion

The demographic data of the patients included in this study corresponded to that in the literature. Although not reaching a statistically significant level, ICU admissions and mortality were mostly associated with blunt injuries. Larger multi-center studies are required to further investigate the incidence of traumatic diaphragmatic injuries (TDI).

## Introduction

Traumatic diaphragmatic injuries (TDIs) involve wounds and diaphragm ruptures; due to thoracoabdominal blunt or penetrating traumas and are associated with other organ injuries in most of the cases [1]. TDIs are uncommon and the true incidence of TDI is difficult to estimate due to the variation in reporting missed or late detected diaphragmatic injuries [2]. These types of injuries result in the disappearance of the thoracoabdominal pressure gradient which allows the abdominal viscera to herniate into the thorax [1,3]. This herniation causes hearing bowel sounds in the thorax, respiratory distress, and bowel obstruction [4]. They are usually missed in up to 60% of the cases in the initial diagnosis, and the most common mechanism of injury was found to be blunt trauma [5]. 

Traumatic injuries of the diaphragm are more commonly seen on the left side. This could possibly be explained by the hepatic protection on the right side and due to the congenital weakness of the left diaphragm [6]. Even though the prevalence is not precisely known because of the injury being missed due to larger or more critical injuries, it was reported that the incidence is between 1% to 7% in blunt trauma cases and 10% to 15% in cases of penetrating trauma [7]. In a case series that included 57 patients with blunt TDI, the diagnosis of diaphragmatic injury was not made until laparotomy was performed in 42% of patients for the other injuries involved in the trauma [8]. In another study that was conducted in the Asir region over a 10-year period, 10 patients had proven traumatic diaphragmatic rupture cases with different mechanisms of injuries. The mortality rate in patients with early diagnosis ranged from 11% to 37%. However, in patients who were recently diagnosed, the mortality rate increased to between 30% to 56%. Therefore, careful clinical examination coupled with accurate interpretation of the symptoms must be made to detect TDIs at the initial phase [9].

In Saudi Arabia, the road traffic accident rate is high with different types of injuries including multiple chest, bone, and abdominal injuries are seen [10]. However, traumatic diaphragmatic injuries are not commonly seen. So far, a small number of studies about the incidence of traumatic diaphragmatic injury have been conducted in Saudi Arabia, which raised the question of our research. Moreover, traumatic diaphragmatic injury is rarely reported nationally and internationally. Therefore, our study’s aim was to investigate the prevalence of traumatic diaphragmatic injury in the thoracoabdominal trauma, discuss the etiological factors, diagnostic investigations, and outcomes in TDIs, and evaluate predictors of mortality in patients who were diagnosed at King Abdulaziz Medical City in Saudi Arabia.

## Materials and methods

This study was designed as an observational retrospective cohort study of adults with traumatic diaphragmatic injuries between the years 2016 and 2020. The study was conducted at King Abdulaziz Medical City (KAMC), Riyadh, Saudi Arabia. The study involved all patients aged 18 years and above diagnosed with traumatic diaphragmatic injury. The subjects were selected in a non-randomized consecutive sampling technique. The BESTCare electronic system was used to get patients’ medical records and extract the demographic data (age, gender, and BMI), surgical data (type of surgery, type of mesh, and operation details), and follow-up data (length of stay, complications, and mortality). Exclusion criteria included all patients aged less than 18. The data were collected into Microsoft Excel to be organized before being analyzed.

Statistical analyses were carried out using Statistical Package for the Social Sciences, version 25.0 (IBM Corp., Armonk, NY). Categorical variables are presented as proportions, and continuous variables as mean ± standard deviation (SD). Pearson χ2 test was used for categorical variables, and an independent t-test was used for continuous variables to investigate the association between predictors and outcomes. A p-value of <0.05 was used to report the statistical significance. The study was granted approval by the Institutional Review Board of King Abdullah International Medical Research Center, Riyadh, Kingdom of Saudi Arabia (approval number IRB/0697/23).

## Results

A total of eight patients were involved in the current study. All demographic data including age, gender, and other factors are illustrated in Table 1. The mean age of the patients was 49 years SD (±22.99) old. Males outnumbered females by 75%. Patients with blunt injuries were admitted 75% more frequently than patients with penetrating injuries by 25%. Among patients admitted to the hospital, motor vehicle accidents were more common than other mechanisms accounting for 50% of the cases. The number of patients admitted on the left side was N = 7, 85% in comparison to the right side, which had only one patient. All patients included in the study survived admission by 100%. Regarding outcomes, most of the patients required ICU admission 85%. Only two patients died by 25%, and the other five patients were alive by 62% (Table 1).

**Table 1:** Frequencies of socio-demographic status TDI: Traumatic diaphragmatic injuries

Variables	Frequencies (8)
Age	Mean 49.13 SD (±22.99)
BMI	Median (IQR) 29.5 (25.5, 32.5)
Gender	
Male	6 (75.0%)
Female	2 (25.0%)
Type of TDI	
Blunt	6 (75.0%)
Penetrating	2 (25.0%)
Immediate	4 (50.0%)
Delayed	4 (50.0%)
Mechanism of Injury	
Motor Vehicle Accident	4 (50.0%)
Pedestrian struck	2 (25.0%)
Stab injury	2 (25.0%)
Site of injury	
Right Side	1 (15.0%)
Left Side	7 (85.0%)
Survival to Admission	
Survived	8 (100%)
Not- survived	0 (0.0%)
ICU admission	
Yes	5 (85.0%)
No	1 (15.0%)
Mortality	
Died	2 (25.0%)
Alive	5 (62.0%)

Table 2 shows the association between the paramedic arrival and predictors and outcomes. According to paramedic-assisted arrival, two patients died, whether immediately or delayed, and there was no significant difference between paramedic arrivals (p-value = 0.7). Pedestrian-struck patients arrived immediately by 50% while patients who had motor vehicle accidents (MVA) arrived delayed by 75% and there is no significant association between paramedic arrivals p-value = 0.2. 

**Table 2:** Association between the paramedic arrival, predictors and outcomes TDI: Traumatic diaphragmatic injury; MVA: Motor vehicle accidents

	Immediate	Delayed	P-value
Gender			
Female	1 (25.0%)	1 (25.0%)	0.7
Male	3 (75.0%)	3 (75.0%)	
Type of TDI			
Blunt	3 (75.0%)	3 (75.0%)	0.7
Penetrating	1 (25.0)	1 (25.0%)	
Mechanism of Injury			
MVA	1 (25.0%)	3 (75.0%)	0.2
Pedestrian Struck	2 (50.0%)	0 (0.0%)	
Stab Injury	1 (25.0%)	1 (25.0%)	
Mortality			
Died	1 (25.0%)	1 (25.0%)	0.7
Alive	3 (75.0%)	2 (75.0%)	

All patients had a lower mortality rate with trauma injuries, either right or left. Eighty-three percent of patients were alive, and there is no significant association between sites of injury and mortality (p-value = 0.2) (Table 3). 

**Table 3:** The association between mortality outcomes and the site of injury

Mortality	Site of injury		P-value
	Right	Left	
Died	1 (100.0%)	1 (16.7%)	0.2
Alive	0 (0.0%)	5 (83.3%)	

Table 4 presents the association between TDI and outcomes. The total number of patients who were admitted to the ICU was five, four of whom had blunt trauma (100%) and one had a penetrating injury (50%); there was no significant association between ICU admission and TDI (p-value = 0.3). The total number of patients who died was two, and both had blunt injuries (40%). Five patients were alive, three of whom had blunt injuries (60%), while two had penetrating injuries (100%), and there was no significant association between mortality and TDI (p-value = 0.4).

**Table 4:** The association between TDI and its outcomes TDI: Traumatic diaphragmatic injury

	Type of TDI			P-value
	Blunt		Penetrating	
ICU admission				
Yes	4 (100.0%)	1 (50.0%)		0.3
No	0 (100.0%)	1 (50.0%)		
Mortality				
Died	2 (40.0)	0 (0.0%)		0.4
Alive	3 (60.0%)	2 (100.0%)		

## Discussion

The demographic data of the patients included in this study corresponded to that in similar studies done in other countries. A retrospective study included 687 patients with diaphragmatic rupture; the mean age reported was 46.1 years, similar to 49.13 in the current study [6]. The majority of the patients were reported to be male (71.9%), similar to 75% in the current study. The same study also reported that blunt trauma outnumbered the penetrating trauma and accounted for 73.5% which is also similar to the current study (75%). It is believed that the predominant site of injury is the left side rather than right-sided injuries [11], and the findings of this study agree with that as well; some owed it to the fact that the liver is less likely to herniate to the chest which, in turn, can be easily missed on imaging [12].

Although not reaching a statistically significant level, ICU admissions and mortality were mostly associated with blunt injuries (p-value = 0.3) (p-value = 0.4). A study done by Fair et al. with a total number of 833,308 cases included showed similar results; patients with blunt traumas had a higher injury severity score (33 ± 14 vs 24 ± 15, p-value < .001), significantly longer ICU length of stay and a higher mortality rate (19.8% vs 8.8%, p-value < .001) as opposed to patients with penetrating injuries [2]. Finally, The diagnosis of traumatic diaphragmatic injuries (TDIs) can be easily missed or delayed and can lead to life-threatening complications. Therefore, a high index of suspicion is required for timely diagnosis.

Study limitations

There are a few limitations to the present study. The primary limitation of this study is the sample size which is relatively small compared with other studies, which is attributed to the rarity of this injury and the nonspecific clinical and radiological findings along with its single-center design.

## Conclusions

The current study concluded that traumatic diaphragmatic injuries (TDIs) are uncommon as only eight cases were detected in our study timeline from 2016 to 2020. Due to the rare appearance of TDIs, the diagnosis can be easily missed or delayed and can lead to life-threatening complications. Further studies with larger sample sizes are recommended to better investigate the incidence and evaluate the predictors of mortality rate.
